# A two-dimensional molecular beacon for mRNA-activated intelligent cancer theranostics†
†Electronic supplementary information (ESI) available. See DOI: 10.1039/c4sc03894k


**DOI:** 10.1039/c4sc03894k

**Published:** 2015-04-08

**Authors:** Dan Wu, Guofen Song, Zhi Li, Tao Zhang, Wei Wei, Muzi Chen, Xuewen He, Nan Ma

**Affiliations:** a The Key Lab of Health Chemistry and Molecular Diagnosis of Suzhou College of Chemistry , Chemical Engineering and Materials Science , Soochow University , 199 Ren'ai Road , Suzhou , 215123 , P. R. China . Email: nan.ma@suda.edu.cn

## Abstract

Ideal theranostics should possess directly correlated imaging and therapy modalities that could be simultaneously activated in the disease site to generate high imaging contrast and therapeutic efficacy with minimal side effects. However, so far it still remains challenging to engineer all these characteristics into a single theranostic probe. Herein, we report a new type of photosensitizer (PS)-derived “two-dimensional” molecular beacon (TMB) that could be specifically activated within tumor cells to exhibit both high imaging contrast and therapeutic efficacy that outperforms conventional photosensitizers for cancer theranostics. The TMB is constructed by integrating a photosensitizer (chlorin e6 (Ce6)), a quantum dot (QD), and a dark quencher (BHQ3) into a hairpin DNA molecule to generate multiple synergistic FRET modes. The imaging modality and therapy modality, which are mediated by FRET between the QD and BHQ3 and FRET between the QD and Ce6 respectively, are interconnected within the TMB and could be simultaneously activated by tumor mRNA molecules. We show that highly effective cancer imaging and therapy could be achieved for cancer cell lines and xenografted tumor models. The reported TMB represents an unprecedented theranostic platform for intelligent cancer theranostics.

## Abstract 2

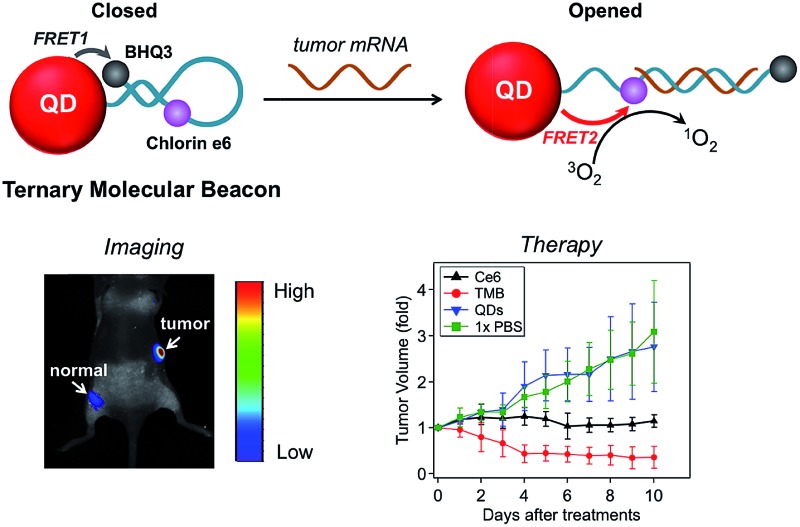
A two-dimensional quantum dot molecular beacon with interconnected imaging and therapy modalities is developed for intelligent cancer theranostics.

## Introduction

Cancer theranostics, an emerging platform to simultaneously assess the disease state of individual patients and tailor the therapeutic treatment based on diagnostic results, holds great promise for personalized medicine.^1^–^3^ Conventional cancer theranostics mainly relies on tumor-targeted co-delivery of imaging and therapeutic agents within the same dose. However, the undesired biodistribution of these theranostic probes can lead to non-specific imaging background and therapeutic side effects.^4^,^5^ Activatable theranostics could provide a valid solution to these drawbacks. Ideally, a smart and effective theranostic probe should fulfill the following criteria: (i) the imaging and therapeutic modalities are specifically activated in the disease site to avoid imaging background and side effects; in particular, it is ideal to activate the probe with endogenous cancer markers to offer the best spatial and temporal control; (ii) the probe should possess both high imaging sensitivity and therapeutic potency; (iii) the imaging and therapeutic modalities need to be correlated in each probe to afford therapeutic capacities with high predictability and controllability. However, so far it still remains challenging to engineer all these characteristics into a single theranostic probe.

Photodynamic therapy (PDT) is an important type of clinically approved therapeutic modality for cancer treatment.^6^–^8^ It is based on light excitation of a photosensitizer (PS) to produce highly reactive singlet oxygen (^1^O_2_) and free radicals that can cause irreversible damage of cellular components and subsequent cell death.^9^–^11^ However, a few bottlenecks of conventional PS molecules including their low extinction coefficient and fluorescence quantum yield (QY), poor photostability, and lack of molecular selectivity have imposed restrictions on their performance as both a therapeutic and an imaging modality.^12^–^14^ To date little success has been achieved to tackle all the above shortcomings of PSs simultaneously, precluding the construction of intelligent theranostic probes based on this important therapeutic modality.

## Results and discussion

**Scheme 1:**
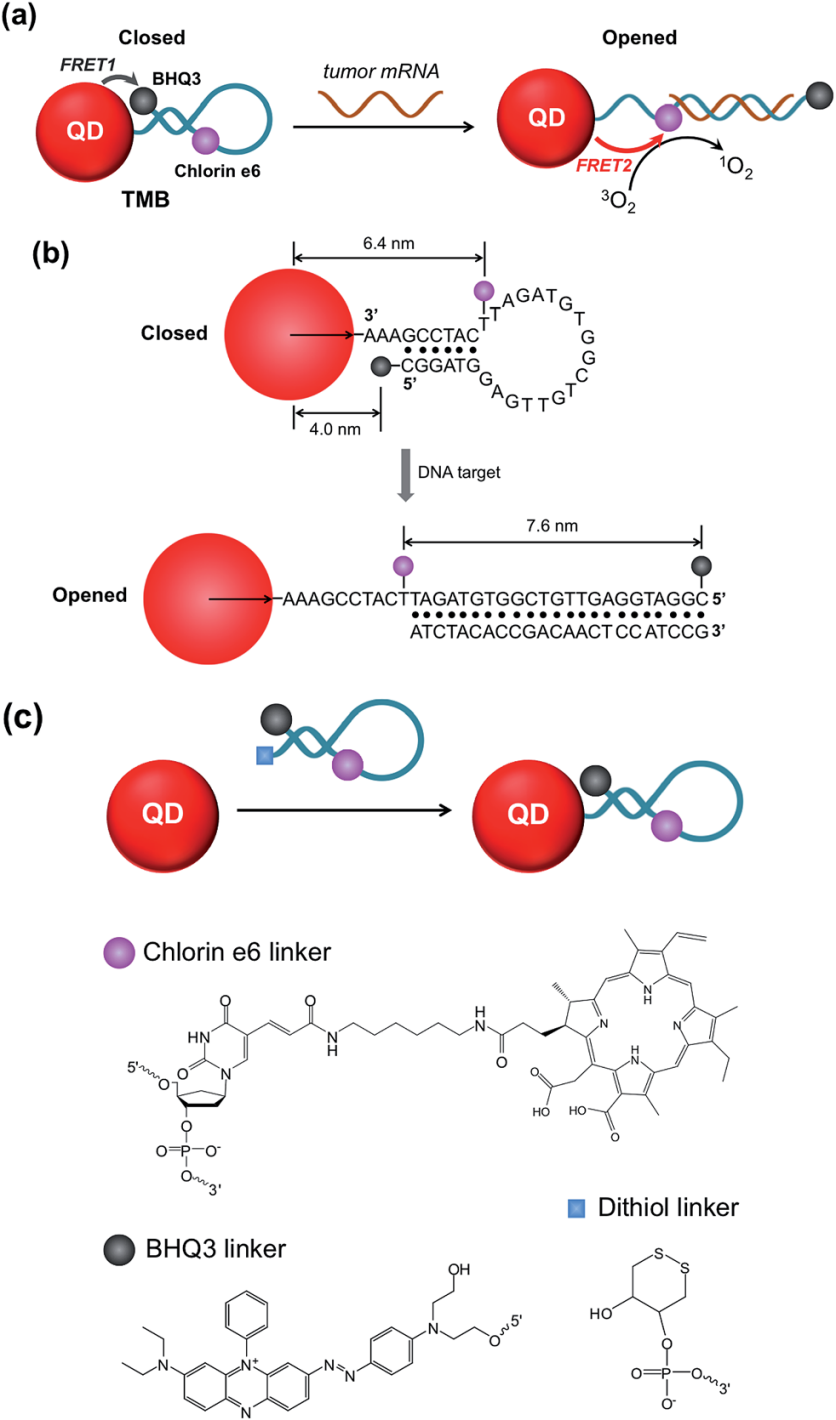
Schematic illustration of the TMB. (a) FRET modes of TMB in the closed and opened forms; (b) hairpin DNA sequence and the separation distance of each FRET pair; (c) chemical structures of Ce6, BHQ3, and dithiol linker.

In this study we sought to develop a new type of PS-derived activatable two-dimensional MB (TMB or 2D-MB) with significantly boosted imaging signal and PDT efficacy for cancer theranostics. Molecular beacons (MBs) are a class of FRET-based hairpin DNA probes that can be opened by specific nucleic acid targets through hybridization and transduce the binding event to an optical signal,^15^ which has offered tremendous opportunities for biosensing and molecular imaging.^16^–^20^ So far all the MBs are constructed on a “one-dimensional” basis – a single adjustable FRET mode controlled by two optical active components tethered to each end of the hairpin DNA. However, this design is only applicable to single functionality activation. Herein, we report for the first time a two-dimensional dual-modal MB containing three functional components with multiple synergistic FRET modes for simultaneous cancer imaging and therapy. Specifically, the TMB is constructed by integrating a photosensitizer (PS) (chlorin e6 (Ce6)), a QD, and a dark quencher (BHQ3) into a hairpin DNA molecule (Scheme 1a). The imaging modality and therapy modality, which are mediated by FRET between the QD and BHQ3 (mode 1) and FRET between the QD and Ce6 (mode 2) respectively, are interconnected within the TMB and could be simultaneously activated by tumor mRNA molecules. Because this beacon contains one donor (QD) and two acceptors (Ce6 and BHQ3), the FRET occurs in two different directions that can be viewed as “two-dimensional”, which is distinct from the traditional one-dimensional molecular beacon. QDs with strong photoluminescence, robust photostability, and large absorption cross-section are used as both robust imaging contrast agents^21^–^31^ and effective light absorbers to sensitize PS molecules.^32^–^34^ BHQ3 is used to maintain the whole system in a dark state before activation. In order to achieve high activatability with minimal background signal, it is critical to precisely tailor the separation distance of each FRET pair within the probe. As shown in Scheme 1b, the QD and BHQ3 are positioned at each end of the hairpin DNA while Ce6 is positioned internally close to the QD. In the closed form the QD PL is efficiently quenched by BHQ3 through FRET mode 1 due to their small separation distance (4.0 nm). When the TMB hybridizes with the tumor mRNA target (Cyclin D1 mRNA), FRET mode 2 becomes active and thus the imaging and therapy modalities are simultaneously activated (Scheme 1a). Based on this design, the imaging and therapeutic modalities are directly correlated within the probe, which could provide high theranostic accuracy down to single-molecule level.

**Fig. 1:**
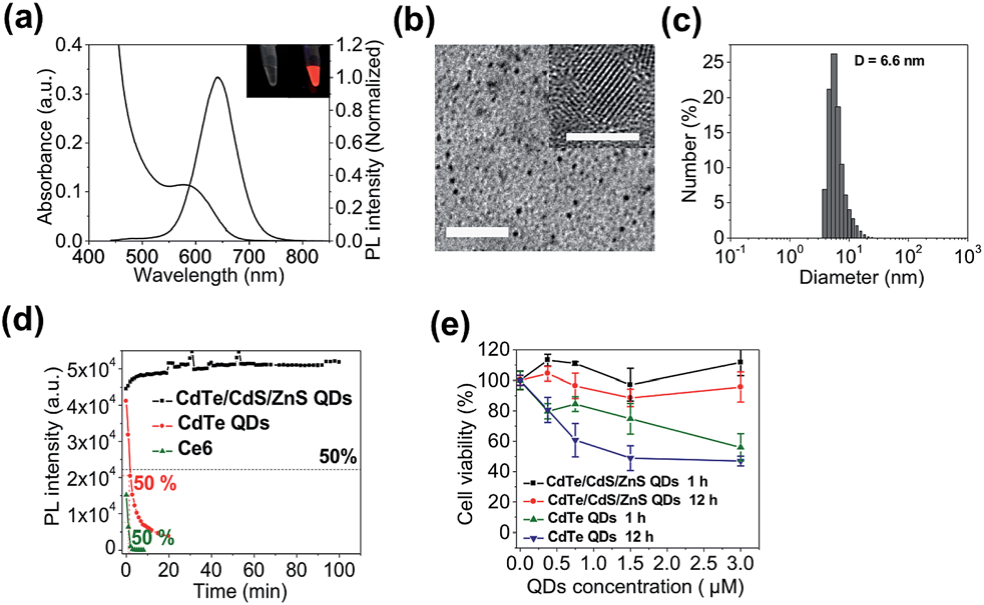
Characterization of CdTe/CdS/ZnS core/shell/shell QDs. (a) Absorption and photoluminescence spectra of CdTe/CdS/ZnS QDs (inset: photographs of CdTe/CdS/ZnS QDs solutions under ambient light (left) and UV light (right)). (b) Low magnification and high-resolution (inset) images of CdTe/CdS/ZnS QDs (scale bars are 50 nm and 5 nm respectively). (c) Hydrodynamic diameter of CdTe/CdS/ZnS QDs measured by DLS. (d) Photostabilities of CdTe/CdS/ZnS QDs, CdTe QDs, and Ce6 molecules excited with a 405 nm laser (110 mW). (e) Cell viabilities of MCF-7 cells incubated with CdTe/CdS/ZnS QDs and CdTe QDs for 1 and 12 hours.

We start with the synthesis of CdTe/CdS/ZnS core/shell/shell QDs with minimal cytotoxicity and robust photostability. Sequential growth of CdS and ZnS shells over the CdTe core results in a stepwise red-shift of the QDs emission peak and enhanced QYs (ESI Fig. S1†). The final product exhibits an emission peak centered at 643 nm (Fig. 1a) and a quantum yield (QY) of 44.7%. Monodisperse nanocrystalline QDs with a mean diameter of 6.1 ± 0.9 nm could be visualized in TEM images (Fig. 1b). The mean hydrodynamic diameter of these QDs is 6.6 nm as determined by dynamic light scattering (DLS) (Fig. 1c). The as-prepared CdTe/CdS/ZnS QDs exhibited robust photostability when continuously excited with a 405 nm laser (110 mW) whereas Ce6 molecules were quickly photobleached under the same conditions (Fig. 1d). Also, these CdTe/CdS/ZnS QDs maintain their stability with minimal fluctuation of PL intensity at various pH values (9.0, 7.4, 5.0) (ESI Fig. S2†). Cytotoxicities of QDs were evaluated using MTT assay. Little effects on cell viabilities were observed for CdTe/CdS/ZnS QDs at all the tested QD concentrations (0–3 μM) whereas CdTe QDs exhibited elevated cytotoxicity at high QD concentrations (Fig. 1e).

**Fig. 2:**
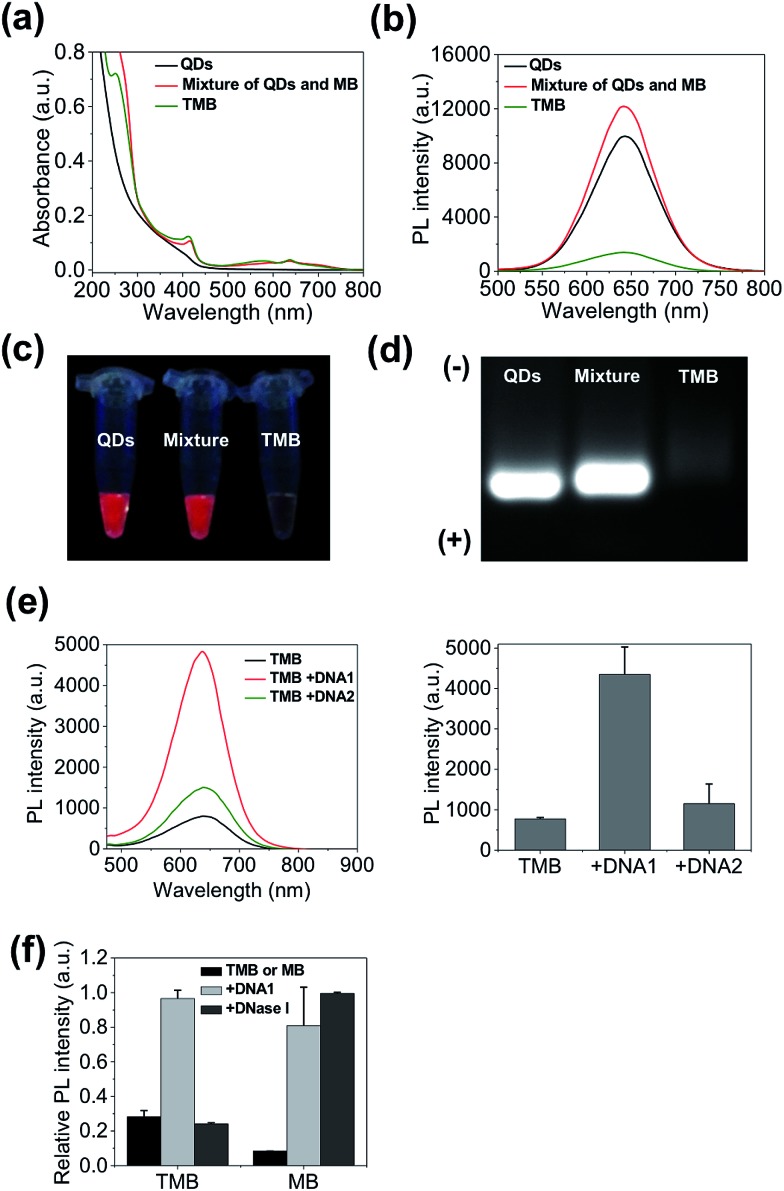
TMB construction and activation. (a) Absorption spectra; (b) photoluminescence spectra; (c) photographs under UV excitation; (d) agarose gel electrophoresis of CdTe/CdS/ZnS QDs, mixture of QDs and MB (without TCEP reduction), and TMB. (e) Activation of TMB with perfect-matched DNA1 and single-base-mismatched DNA2 measured by photoluminescence spectra. (f) Digestion of TMB and MB with DNase I measured by the PL intensities of the probes. DNA1-activated TMB and MB are used as controls.

The QD-sensitized TMB was constructed in two steps. First, a hairpin DNA molecule modified with a BHQ3 molecule (5′ position), a Ce6 molecule (internal amino modifier C6 dT), and a dithiol group (3′ position) was synthesized (Scheme 1c). Second, the dithiol group of the modified DNA was reduced with tris(2-carboxyethyl)phosphine (TCEP) and then directly attached to the surface of the CdTe/CdS/ZnS QDs (see Experimental section, ESI,† for more details). This conjugation method would allow a high number of DNA molecules to be attached to each QD to acquire optimal FRET efficiency. As shown in Fig. 2a, the absorption spectrum of the purified QD–DNA conjugate contains absorption features of both QDs and DNA molecules, indicating successful conjugation of DNA to QDs. The average number of DNA molecules attached to each QD is about 10 as determined by UV-Vis spectroscopy. FRET parameters (spectral overlap *J*, Förster distance *R*_0_, and FRET efficiency *E*) of each FRET pair are calculated and summarized in Table 1. Pronounced decrease of QDs fluorescence lifetime was observed after conjugation of the QDs with Ce6 molecules (ESI Fig. S3†), confirming efficient FRET between the QD and Ce6. On the contrary, only marginal difference in QDs fluorescence lifetime was observed between the QDs in argon-purged solution and O_2_-saturated solution (ESI Fig. S3†), indicating that direct sensitization of O_2_ with QDs is negligible. DNA conjugation leads to efficient quenching of QDs PL by BHQ3 (Fig. 2b and c). Conjugation was further monitored using agarose gel electrophoresis. The QD–DNA conjugate exhibited a faint band with retarded mobility in the gel in comparison with unmodified QDs and the mixture of QDs and DNA (without TCEP reduction) (Fig. 2d), revealing increased overall size of the QDs and pronounced PL quenching after DNA conjugation. The activatability of the TMB was evaluated using two DNA sequences – a perfectly matched target (DNA1) and a single-base mismatched target (DNA2). As shown in Fig. 2e, the QD PL could be significantly recovered when treated with DNA1. In contrast, only marginal PL enhancement was detected for the TMB treated with DNA2. These results suggest that the activation of the TMB is sequence-specific and could discriminate single mutations. The stability of the TMB against nuclease degradation was also evaluated. No PL increase was observed after treating the TMB with DNase I (Fig. 2f), indicating that the DNA attached to the QDs remained intact. This is presumably because of inhibitory effects of steric hindrance caused by densely loaded DNA on the nanoparticle surface. In contrast, Ce6 and BHQ3-modified hairpin DNA without QD conjugation (MB) exhibited significant enhancement of Ce6 fluorescence under the same treatment (Fig. 2f), indicating efficient digestion of free hairpin DNA by DNase I. These results are further confirmed by denaturing PAGE (ESI Fig. S4†). The DNA of TMB remained intact after DNase I treatment whereas the free MB was completely digested by DNase I. Although the signal-to-background (*S*/*B*) ratio of MB (9.7) is higher than TMB (3.4), the MB is not suitable for *in vivo* studies because of its instability against nuclease digestion. While the activation kinetics of TMB could be accelerated by decreasing the number of conjugated oligonucleotides (ESI Fig. S5†), quenching of QD PL and nuclease protection became less efficient as the number of conjugated oligonucleotides on each QD decreased (Fig. S6†).

**Table 1:** Summary of spectral overlap *J*, Förster distance *R*_0_, and FRET efficiency *E* of each FRET pair of TMB. See Experimental section for detailed calculations (ESI†)

FRET pair	*J* (cm^3^ M^–1^)	*R*_0_ (nm)	*r* (close) (nm)	*r* (open) (nm)	*E* (close)	*E* (open)
QD–BHQ3 (*n* = 10)	5.04 × 10^–14^	4.0	4.0	14.0	91%	—
QD–Ce6 (*n* = 10)	3.44 × 10^–13^	5.5	6.4	6.4	—	80%
Ce6–BHQ3 (*n* = 1)	6.94 × 10^–14^	3.4	2.0	7.6	96%	0.79%

**Fig. 3:**
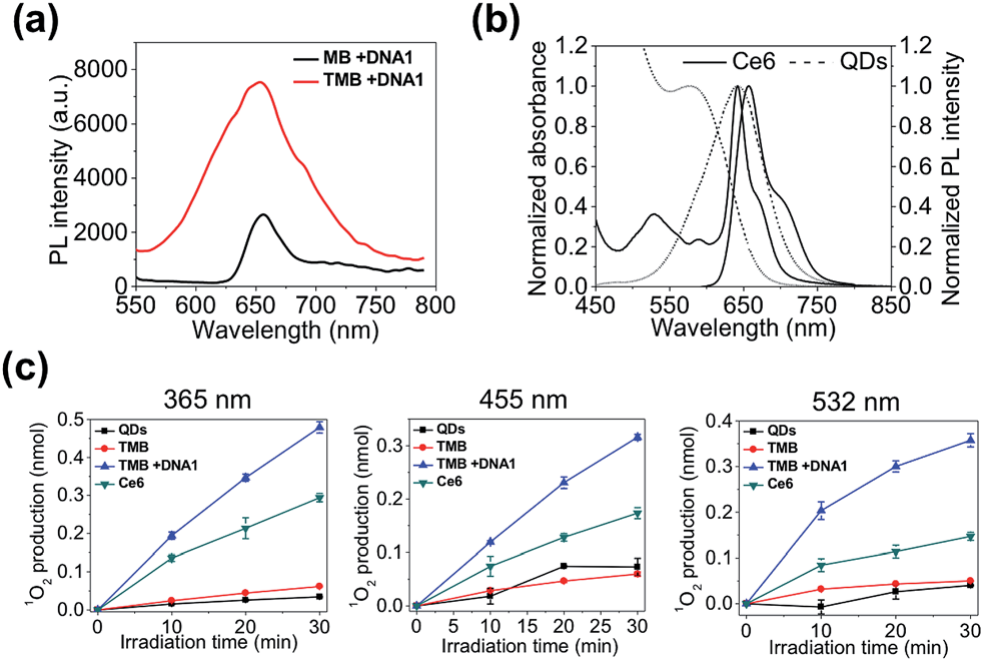
*In vitro* evaluation of theranostic efficacy of TMB. (a) Photoluminescence spectra of DNA1-activated TMB and MB (same Ce6 concentration) excited at 405 nm. (b) Normalized absorption spectra and photoluminescence spectra of CdTe/CdS/ZnS QDs and Ce6 molecules. (c) Quantification of ^1^O_2_ generation by QDs, TMB, DNA1-activated TMB, and free Ce6 (same Ce6 concentration) irradiated at 365 nm, 455 nm, and 532 nm for 10, 20, and 30 minutes.

Next, we evaluated the theranostic efficacy of the TMB *in vitro*. Ce6 molecule has an excitation maximum at 404 nm (Fig. S7†). As shown in Fig. 3a, the emission signal (integrated emission peak) of the DNA1-activated TMB is 4.8-fold higher than that of the DNA1-activated Ce6/BHQ3-modifed hairpin DNA (MB) with the same Ce6 concentration, which could be attributed to a larger extinction coefficient and higher QY of QDs than Ce6 molecules. It is noteworthy that only one emission peak was observed for TMB because of the overlapping of the QDs PL peak with the Ce6 emission peak (Fig. 3b). A ^1^O_2_ probe – 9,10-anthracenediyl-bis(methylene) dimalonic acid (ABMD) was used to quantitate the amount of ^1^O_2_ generated by the activated TMB and free Ce6 molecules (same Ce6 concentration). The two probes were continuously illuminated with light at three different wavelengths (365 nm, 455 nm, 532 nm) for different durations, and the amounts of ^1^O_2_ produced in each reaction were measured according to the decrease of ABMD absorption at 400 nm. As shown in Fig. 3c, the amounts of ^1^O_2_ generated by the activated TMB are consistently higher than that of free Ce6 molecules under all the tested conditions, which could be attributed to efficient light absorption by QDs and subsequent efficient energy transfer to Ce6.

**Fig. 4:**
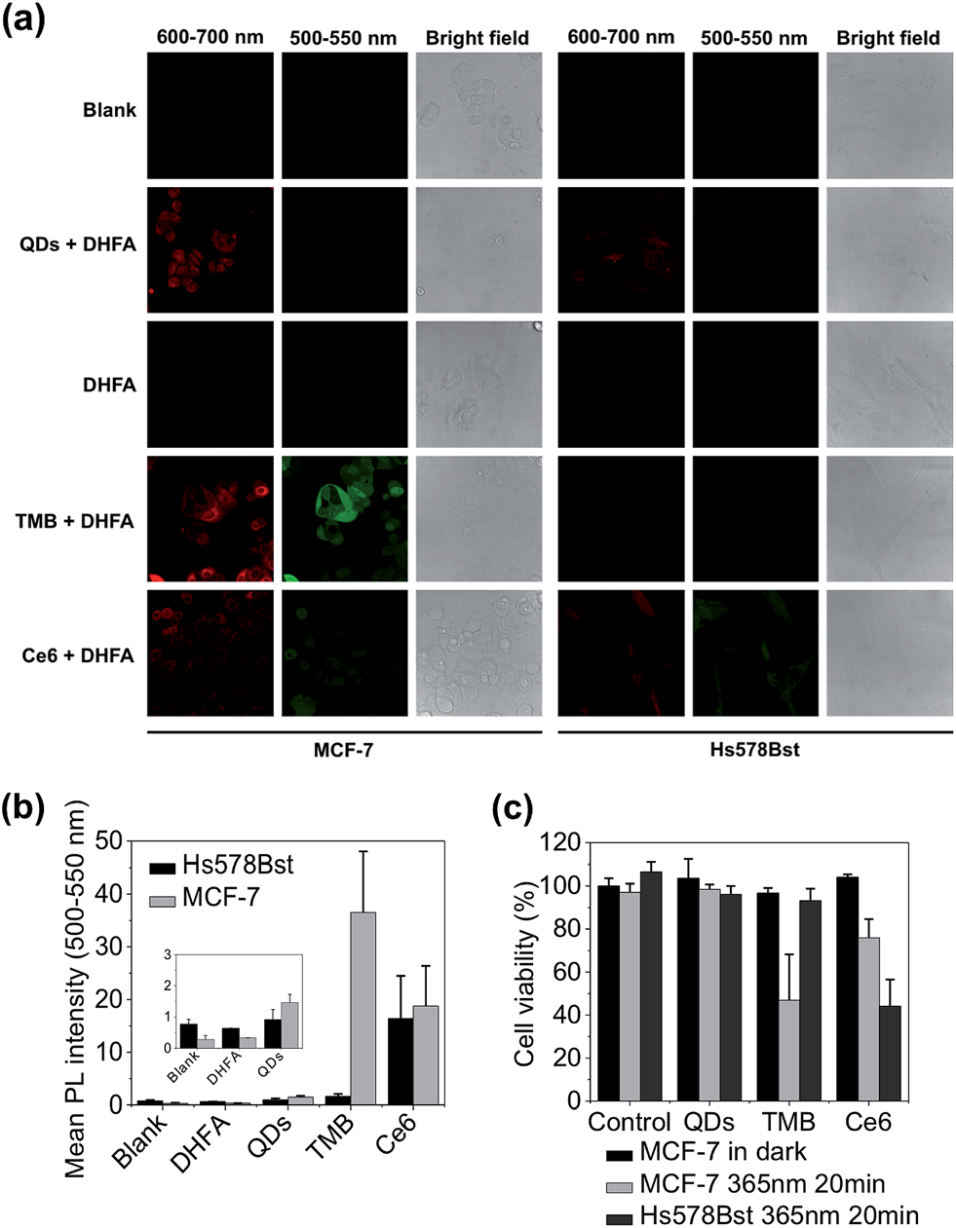
Evaluation of theranostic efficacy of TMB with live cancer cells. (a) Confocal microscopy images of MCF-7 and Hs578Bst cells incubated with QDs, TMB, and Ce6 and further treated with DHFA for intracellular ^1^O_2_ detection. Fluorescence signals (red) of each probe were collected between 600 nm and 700 nm with 405 nm excitation. DHFA fluorescence (green) was collected between 500 nm and 550 nm with 488 nm excitation. (b) Mean DHLA PL intensities of MCF-7 and Hs578Bst cells treated with QDs, TMB, and Ce6. (c) Cell viabilities of MCF-7 and Hs578Bst cells incubated with QDs, TMB, and Ce6. Cells were incubated with each probe for 3 hours. PDT was implemented with 365 nm UV light irradiation for 20 min.

The theranostic efficacy of TMB was then evaluated at the cell level using MCF-7 breast cell line containing overexpressed cyclin D1 mRNA. A normal human mammary epithelial cell line Hs578Bst with minimal expression level of cyclin D1 mRNA was used as the control cell line. An intracellular ^1^O_2_ sensor – 2,7-dichlorodihydrofluorescein diacetate (DHFA) that emits green fluorescence upon reacting with ^1^O_2_ is used to detect ^1^O_2_ generation inside the cells. The cells were first incubated with each probe (QDs, TMB, Ce6) for 3 hours at 37 °C and then further incubated for 12 hours at 37 °C in fresh media. Next, the cells were incubated with DHFA for 30 min followed by illumination with 365 nm UV light for 10 min. Cell images were acquired on a confocal microscope. As shown in Fig. 4a, unmodified QDs were internalized into both cell lines after prolonged incubation as indicated by the red fluorescence inside the cells. After incubation with TMB, only MCF-7 cells but not Hs578Bst cells exhibited pronounced red fluorescence, indicating specific intracellular activation of the TMB in MCF-7 cells. Confocal co-localization study reveals that the QD–DNA nanoprobes were endocytosed and located in late endosome and lysosome of MCF-7 cells at early stage but escaped from the endosome and lysosome after prolonged incubation (ESI Fig. S8†). Meanwhile, the activated TMB probe produced high levels of ^1^O_2_ in MCF-7 cells as revealed by the strong green fluorescence from DHFA (Fig. 4a and b). In contrast, no ^1^O_2_ was detected for Hs578Bst cells treated with TMB. Additionally, both cells incubated with Ce6 molecules exhibited weak Ce6 and DHFA fluorescence, indicating that Ce6 could enter cells and generate a moderate amount of ^1^O_2_ without selectivity. Therefore, the TMB could provide high specificity, strong fluorescence, and high ^1^O_2_ yield that are unavailable for free Ce6 molecules. Cell viabilities were measured using MTT assay. As shown in Fig. 4c, the TMB could effectively kill MCF-7 cells but exhibited no adverse effects on Hs578Bst cells. In contrast, Ce6 exhibited moderate toxicity to both MCF-7 cells and Hs578Bst cells.

**Fig. 5:**
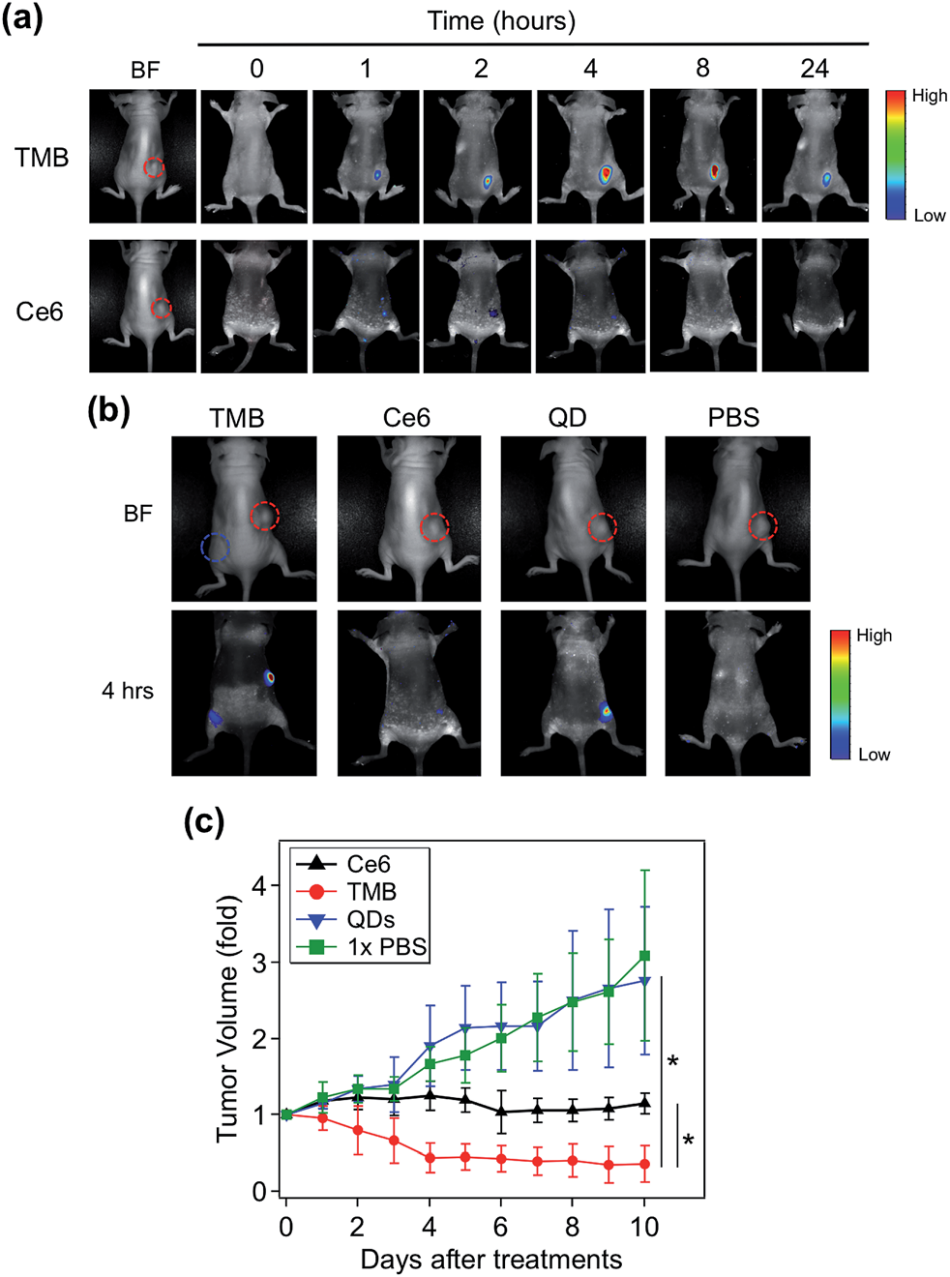
TMB-based cancer theranostics for xenografted tumor models (MDA-MB-231 tumor). (a) Whole body bright field and fluorescence images of tumor-bearing mice injected with TMB and Ce6 (same Ce6 concentration) as a function of time. The images were recorded pre-injection (0 hour) and post-injection at different time points (1, 2, 4, 8, 24 hours). Images were acquired with 455 nm excitation and the emission signals were collected between 610 and 750 nm (xenografted tumors are highlighted by red dashed circles). (b) Whole body bright field and fluorescence images of tumor-bearing mice injected with TMB, Ce6, QDs, and 1× PBS. The fluorescence images were taken 4 hours after injection (xenografted tumors are highlighted by red dashed circles; subcutaneous injection site is highlighted by the blue dashed circle). (c) Growth curves of tumors injected with TMB, Ce6, QDs, and 1× PBS followed by PDT treatment (*p < 0.05 indicating significant difference between TMB and Ce6 groups and significant difference between TMB and QDs groups).

TMB-based *in vivo* cancer theranostics were assessed with xenografted tumor models (MDA-MB-231) of nude mice. TMB and Ce6 (same Ce6 concentration) were injected intratumorally and the fluorescence images of each tumor-bearing mouse were recorded on a Maestro *In Vivo* Imaging System at different time points. As shown in Fig. 5a, TMB were gradually activated within the tumor where the PL intensity reached plateau after 4 hours and declined after 24 hours. In contrast, the tumor injected with the same dose of free Ce6 only exhibited marginal fluorescence at early time points which was quickly diminished thereafter. This result could be attributed to both the weak fluorescence and poor photostability of Ce6 molecules. Similar results were observed for MCF-7 tumor-bearing mice (ESI Fig. S9†). To further confirm that the strong PL signal of TMB in the tumor site is due to specific activation, we injected the same amount of TMB subcutaneously and intratumorally into two sites of the same mouse. As shown in Fig. 5b, the tumor site exhibited strong PL whereas the normal site only exhibited weak PL after 4 hours, confirming that the TMB could be specifically activated *in vivo*. To evaluate the therapeutic efficacy of the TMB, tumors were injected with TMB, Ce6, unmodified QDs, and 1× PBS respectively and then irradiated with 455 nm light to promote ^1^O_2_ generation. Changes of tumor volumes were recorded daily for 10 days post treatment. As shown in Fig. 5c, TMB could cause effective tumor regression after PDT with the average tumor volume shrunk by 63% after 10 days. Free Ce6 could suppress tumor growth whereas the average tumor volume remained little changed. Unmodified QDs have little effect on tumor growth. Biodistribution measured by ICP-OES shows that the intravenously injected QD–DNA probes were mainly accumulated in liver, spleen, lung, kidney, and tumor (ESI Fig. S10†). Histological analysis reveals that these QD nanoprobes were non-toxic to the major organs (ESI Fig. S11†). Taken together, the TMB shows several advantages over free Ce6 molecules: (i) TMB exhibits both higher imaging sensitivity and therapeutic efficacy than Ce6 owing to QD-based sensitization; (ii) the molecular specificity of TMB allows PDT to be performed with high spatial resolution to avoid adverse effects on surrounding normal cells; (iii) the TMB could serve as a useful platform for tumor mRNA imaging-guided personalized cancer treatment.

## Conclusion

In conclusion, we have constructed a new type of two-dimensional dual-modal MB for mRNA-activated cancer theranostics. To the best of our knowledge, this is the first example of a higher-dimensional MB containing interconnected multiple FRET modes for advanced biomedical applications. Traditional MB probes suffer from low photostability, weak fluorescence, moderate singlet oxygen production, and low stability against nuclease and are therefore incompetent for *in vivo* studies. We have demonstrated that the TMB could overcome these disadvantages and provide high imaging sensitivity and therapeutic efficacy. Also, the TMB presents an unprecedented theranostic platform with high molecular specificity and direct-correlated imaging and therapeutic modalities, which will pave the way for intelligent cancer theranostics. In particular, the TMB would allow identification of cancerous tissues with high confidence and implementation of PDT with molecular accuracy. Additionally, this theranostic TMB could be extended to other cancer types and diseases owing to the large number of disease-related mRNA molecules identified to date.

## Supplementary Material

Supplementary information
