# Supplementary material for: A two-dimensional molecular beacon for mRNA-activated intelligent cancer theranostics
Source: Chem Sci. 2015 Apr 8;6(7):3839–44. doi: 10.1039/c4sc03894k (PMC5707452; doi:10.1039/c4sc03894k)
Supplement: Supplementary file 1 [file SC-006-C4SC03894K-s001.pdf]

Electronic Supplementary Material (ESI) for Chemical Science.
This journal is © The Royal Society of Chemistry 2015

Electronic Supplementary Information

A Two-Dimensional Molecular Beacon For mRNA-Activated Intelligent Cancer Theranostics

Dan Wu[a§], *Guofen Song*[a§], *Zhi Li*[a], *Tao Zhang*[a], *Wei Wei*[a], *Muzi Chen*[a], *Xuewen He*[a], *and Nan Ma*[a]*

[a]The Key Lab of Health Chemistry and Molecular Diagnosis of Suzhou
College of Chemistry, Chemical Engineering and Materials Science
Soochow University
199 Ren'ai Road, Suzhou, 215123, P. R. China
E-mail: nan.ma@suda.edu.cn
[§]These authors contributed equally to this work

Experimental Section

Materials.

Te powder (99.997%), L-glutathione (reduced) (99%), sodium borohydride ($NaBH_4$, 98.0%), thiourea (99%), zinc acetate dihydrate (99.0%), rhodamine 6G, and 9,10-anthracenediyl-bis(methylene) dimalonic acid (ABMD) were purchased from Sigma-Aldrich. Cadmium chloride ($CdCl_2$, 99.996%) was purchased from Alfa Aesar. All chemicals were used without further purification. MCF-7 and Hs578Bst cells were purchased from Cell Resource Center of Shanghai Institutes for Biological Sciences. MDA-MB-231 cell line was kindly provided by Cold Spring Biotech Corp.. Female athymic nude mice were purchased from Shanghai SLAC Laboratory Animal Co. Ltd (Shanghai, China). Dulbecco's Modified Eagle's Medium (DMEM), fetal bovine serum (FBS), 0.25% trypsin/EDTA, and phosphate buffered saline (PBS) were purchased from Hyclone. Dulbecco phosphate buffered saline (DPBS) containing calcium and magnesium was purchased from Gibco. Water used in all the studies

was purified with a Milli-Q water purification system. All other reagents and solvents were of analytical grade. All the DNA molecules were synthesized and HPLC purified by Takara Biotechnology Co., Ltd (Dalian, China).

Sequences of MB and DNA targets.

Modified hairpin DNA: 5'-BHQ3-CGGATGGAGTTGTCGGTGTAGAT(Chlorin e6-CONHC6 dT) CATCCGAAA-dual SH-3'

DNA1 (perfectly matched DNA target): 5'-A TCT ACA CCG ACA ACT CCA TCC G-3'

DNA2 (single-base mismatched DNA target): 5'-A TCT ACA CCG A***G***A ACT CCA TCC G-3'

(Sequence of cyclin D1 gene: 5'-AGA AGC TGT GCA TCT ACA CCG ACA ACT CCA TCC GGC-3')

([a]Underlined letters represent the stem sequence; [b]Letters in bold italic represent the mismatched base.)

Synthesis of CdTe/CdS/ZnS core/shell/shell QDs.

Synthesis of CdTe QDs: First, a NaHTe solution was prepared by mixing Te powder (0.5 mmol) with $NaBH_4$ (2 mmol) in 1 mL of degassed deionized water in an eppendorf tube. A needle was inserted into the capped tube to release the pressure, and the solution was maintained for 40 min at 60 °C. The Cd^{2+} precursor solution was prepared by dissolving GSH (0.125 mmol) and $CdCl_2$ (0.05 mmol) in 20 mL of deionized water. The solution pH was adjusted to 8.5 with 1 M NaOH solution. The NaHTe solution (25 μL) was injected into N_2 saturated Cd^{2+} precursor solution with a molar ratio of 4:1:10 for Cd^{2+}:NaHTe:GSH. After injection, the color of the solution immediately changed from colorless to yellow. The reaction mixture was heated at 100°C, and aliquots of the reaction mixture (0.2

mL) were collected and characterized every 10 minutes until the QD emission maximum reached 535 nm, after which the reaction was quenched quickly by cooling down to 0 °C in an ice-bath.

Synthesis of CdTe/CdS core/shell QDs: First, the CdTe core nanocrystals were purified and resuspended in 25 mL of degassed water, and the pH of the solution was adjusted to 10.5. The Cd^{2+} precursor solution was prepared to include 30 mM $CdCl_2$, 75 mM GSH, and 25 mM thiourea. Assuming that one CdS monolayer adds 0.335 nm to the radius of the core, precise amounts of the Cd^{2+} and thiourea solutions were injected to form the first monolayer, with a separate addition step for the second monolayer. The temperature of each reaction mixture was held at 90 °C for 60 min before samples were collected for subsequent purification and characterization.

Synthesis of CdTe/CdS/ZnS core/shell/shell QDs: First, Zn^{2+} precursor solution was prepared by dissolving GSH (0.2 mmol) and $Zn(OAc)_2$ (0.1 mmol) in 25 mL of deionized water, with subsequent adjustment of the solution pH to ~7. Next, 0.025 μmol purified CdTe/CdS nanocrystals and 0.1 mmol thiourea were added to Zn^{2+} precursor solution, and the pH was adjusted to 11. The molar ratio of Zn^{2+}/thiourea/GSH in the reaction mixture was 1:1:2. The reaction mixture was heated at 90 °C, and aliquots of the mixture (0.2 mL) were collected at various time points for characterization. The reaction was subsequently quenched in an ice bath. The resulting QDs were purified by 2-propanol precipitation and were resuspended in deionized water (2 mL) for characterization.

The concentration of CdTe QDs was calculated according to a method reported in *Chem. Mater.* **2003**, 15, 2854-2860. The concentrations of CdTe/CdS QDs and CdTe/CdS/ZnS QDs are directly derived from the CdTe core concentration.

Optical characterization.

Photoluminescence spectra were recorded on a fiber fluorescence spectrophotometer (AvaSpec-ULS2048-USB2) equipped with a 405 nm laser (110 mW) as excitation light source. Absorption spectra were recorded on a UV-Vis spectrophotometer (Agilent 8453). Excitation spectrum of Ce6 was recorded on an Edinburgh FLS920 steady state & time-resolved fluorescence spectrometer.

TEM characterization.

A few drops of each QD sample were dispersed onto a 3 mm copper grid covered with a continuous carbon film and were dried at room temperature. Low magnification and high resolution TEM characterization was performed on a FEI Tecnai G20 and F20 transmission electron microscope operating at 185 kV and 200 kV, respectively.

Dynamic light scattering.

Dynamic light scattering (DLS) measurements were performed on a Zetasizer Nano ZS90 (Malvern) with 90° scattering angle and a He-Ne laser.

Quantum yield determination.

The QY is calculated according to the equation below:

$$\Phi_x = \Phi_{st} (I_x/I_{st}) (\eta_x^2/\eta_{st}^2) (A_{st}/A_x)$$

Where Φ is the quantum yield, I is the measured integrated emission intensity, η is the refractive index of the solvent, and A is the optical density. The subscript "*st*" refers to standard with known quantum

yield and "*x*" refers to the QD sample. Rhodamine 6G (QY = 95% in ethanol) was chosen as the standard.

Photostability measurements of CdTe/CdS/ZnS QDs, CdTe QDs, and Ce6.

CdTe/CdS/ZnS QDs, CdTe QDs, and Ce6 solutions were continuously excited with a 405 nm laser (110 mW) and the fluorescence spectra were record at different time points.

Stability of CdTe/CdS/ZnS QDs in buffer solutions with different pH values

Three aliquots of as-prepared CdTe/CdS/ZnS QDs (1.45 μM) were purified using a Microsep™ Advance Centrifugal Device (YM-30, Pall Corporation) by centrifugation at 12000 rpm for 10 min. The purified QDs were then diluted in 0.2 M HAc-NaAc buffer (pH = 5.0), 0.2 M Tris-HCl buffer (pH = 7.4), and 0.2 M NH_4HCO_3-$(NH_4)_2CO_3$ buffer (pH = 9.0) respectively. Aliquot solutions were taken out at each time point to measure the PL spectra.

Conjugation of QDs with dual-thiol-modified hairpin DNA.

Before conjugation, the dual-thiol-modified hairpin DNA (50 μM) were activated by 75 μM TCEP in 50 mM PBS (pH 7.4) for 2 h at room temperature in a glove box to reduce the disulfide bond. The as-prepared CdTe/CdS/ZnS QDs were purified by 2-propanol precipitation (1:3 v/v) and redispersed in water. The purified QDs were mixed with activated dual-thiol-DNA with a molar ratio of 1:10. The mixture was then incubated at room temperature for 16 h, aged for another 12 h in the presence of 0.2 M NaCl, and finally purified using a Microsep™ Advance Centrifugal Device (YM-30, Pall Corporation) by centrifugation at 12 000 rpm for 10 min.

FRET Analysis.

The donor emission spectrum and the acceptor absorption spectrum were used to calculate the spectral overlap integral:

$$J = \int F_D(\lambda)\ \varepsilon_A(\lambda)\lambda^4\ d\lambda$$

where F_D is the wavelength dependent donor emission spectrum normalized to an area of 1, ε_A is the extinction coefficient spectrum of the acceptor in units of $M^{-1}cm^{-1}$, and λ is the wavelength in cm. The overlap integral is used to calculate the Förster distance, R_0, using the equation:

$$R_0 = 9.78\times10^3\ (\kappa^2 Q_D J\ \eta^{-4})^{1/6}$$

where κ^2 is the dipole orientation factor, assumed to be 2/3, Q_D is the quantum yield of the donor, and η is the refractive index of the medium ($\eta(H_2O)=1.333$).

The average number of acceptors per donor, n, as determined by absorbance spectroscopy, was taken into account. In the case where one donor species can interact with several acceptors the energy transfer efficiency can be expressed as:

$$E = nR_0^6/(nR_0^6+r^6)$$

where r is the separation distance between the donor and the acceptor.

Time-resolved fluorescence spectroscopy

40 nM QDs or QDs-DNA-Ce6 conjugates solution (DNA: 5'-(Ce6)TCATCCGAAA(Dual SH)-3') was purged with argon for 10 min, and the time-resolved fluorescence decay curve was recorded on a HORIB-FM-2015 Spectrofluorometer using 370 nm laser as the excitation source, then the QDs solution was oxygenated by bubbling 5 mL of air using a syringe, and the time-resolved fluorescence decay curve was recorded.

In vitro activation of TMB by DNA target.

TMB (0.1 μM QDs) in 1×DPBS (pH 7.4) was incubated with the perfectly matched DNA target (DNA1) or the single-base-mismatched DNA target (DNA2) (3 μM) for 12 hours. After incubation, the fluorescence was recorded on a fluorescence spectrophotometer.

Nuclease Assay.

Three aliquots of TMB or MB were diluted in 300 μL of 1× reaction buffer (10 mM Tris-HCl (pH 7.5), 2.5 mM $MgCl_2$, 0.1 mM $CaCl_2$) and placed in a 96-well plate at 37 ºC. The concentrations of MB and TMB are 200 nM and 20 nM, respectively. 0.5 μL of DNase I in reaction buffer (1 U/μL) was added to one group and 1 μM of Cyclin D1 DNA target (DNA1) was added to another group and incubated for 12 h at 37 ºC. The fluorescence intensities of TMB (645 nm) and MB (667 nm) were measured under 405 nm excitation.

Denaturing PAGE was performed to further confirm that the DNA attached to QDs remains intact after nuclease treatment. 20 μM MB and 2 μM TMB were treated with 5 μL of DNase I in reaction buffer (1 U/μL) in a total volume of 10 μL and incubated for 12 h at 37 ºC. Afterwards the samples was mixed with 5 μL HCl (pH=3) to digest QDs and the pH was adjusted to 7.4 with Tris-HCl (20 mM). MB without nuclese treatment was used as a negative control.

$^1O_2$ quantification.

Disodium of 9,10-anthracenediyl-bis(methylene) dimalonic acid (ABMD) was employed as a probe to assess $^1O_2$ generation. ABMD molecules can react with $^1O_2$ to yield an endoperoxide, which caused a decrease in the intensity of ABMD absorption. The decreased intensity of ABMD is relative to the quantities of $^1O_2$. Although the absorption spectra of ABMD and Ce6 were overlapped, they

could be clearly distinguished due to the characteristic vibronic peaks of ABMD at 360 nm, 380 nm and 400 nm, respectively. The TMB (0.1 μM QDs) was incubated with the perfectly matched DNA target (DNA1) (3 μM) in 1× DPBS (pH=7.2) for 12 h. Then ABMD (50 μM) was added into the above solution. The mixture was oxygenated by bubbling 2 mL of air using a syringe. The photo-oxidation of ABMD was monitored for 30 min under irradiation with three different light sources (365 nm, 455 nm, 532 nm). Absorption spectra were recorded and 2 mL more air was bubbled in the solution every 10 min. $^1O_2$ generation by QDs and Ce6 was also evaluated under the same conditions. Decrease of ABMD absorbance at 400 nm was used to calculate $^1O_2$ quantities. The number of moles of $^1O_2$ produced was determined as follows (*J. Phys. Chem. C* **2012**, 116, 9334−9342):

$$A(t) = A_{\mathrm{TMB}}(t) + A_{\mathrm{ABMD}}(t)$$

where $A(t)$, $A_{\mathrm{TMB}}(t)$, and $A_{\mathrm{ABMD}}(t)$ represent the total absorbance, the absorbance of TMB, and the absorbance of ABMD, respectively, at time t of the TMB/ABMD mixture. $A_{\mathrm{TMB}}(t)$ is determined using the pure TMB solution. The number of moles of $^1O_2$ produced after a cumulated irradiation time t is given by

$$\Delta n(^1O_2)_{\mathrm{produced}} = \Delta n(\mathrm{ABMD})_{\mathrm{consumed}} = [(A_{\mathrm{ABMD}}(0) - A_{\mathrm{ABMD}}(\mathrm{t})) / A_{\mathrm{ABMD}}(0)] \times n(\mathrm{ABMD}, 0)$$

where n(ABMD, 0) refers to the initial amount of ABMD probe.

Cell culture.

Breast cancer cell line MCF-7 was cultured in 25 cm^2 cell culture flasks with vent caps (Corning) in DMEM supplemented with 10% FBS, 0.01 mg/mL insulin, and 1% antibiotics penicillin/streptomycin (100 U/ml). Normal immortalized human mammary epithelial cell line Hs578Bst was cultured in DMEM/F12 supplemented with 10% FBS and 30 ng/ml EGF. MDA-MB-231 cell line was cultured in DMEM supplemented with 10% FBS. They were all maintained in a

humidified incubator at 37 °C containing CO_2 (5%). Cells that had been grown to subconfluence were dissociated from the surface with a solution of 0.25% trypsin/EDTA. Then aliquots of cells were seeded into an 8-well chamber slide (Lab-Tek) and grown overnight in FBS-containing cell media before experiments.

Confocal laser scanning microscopy.

MCF-7 and Hs578Bst cells were plated on 8-well chamber slides and grown for 24 hours. Cells were first incubated with 50 nM QDs, 50 nM TMB, 500 nM Ce6 in serum-free cell media for 3 hours at 37 °C. Subsequently the serum-free cell media was replaced by fresh FBS-containing cell media and the cells were further incubated for 12 h. Then all cells were washed twice with 1× PBS and incubated with the fluorescent $^1O_2$ probe 2,7-dichlorodihydrofluorescein diacetate (DHFA, 10 μM) in serum-free cell media for 30 min. When taken up by cells, the acetate groups present on DHFA are cleaved by esterase enzymes leaving it susceptible to oxidation by singlet oxygen, the reduced form of hydrolyzed DHFA is non-fluorescent and the oxidized form of hydrolyzed DHFA is fluorescent. Afterwards cells were washed 3 times with 1× PBS and kept in fresh FBS-containing cell media under irradiation of 365 nm UV light for 10 min. Cell images were acquired on a Leica TCS SP5 II confocal laser scanning microscope using a 63× oil immersion objective. QDs, TMB, and Ce6 were excited at 405 nm and the emission signals were collected between 600-700 nm; DHFA were excited at 488 nm and the emission signals were collected between 500-550 nm. Mean fluorescence intensities of cells were quantified using Leica application suite and ten fields of view including approximately 100 cells were analyzed.

Co-localization experiments

MCF-7 cells were first incubated with 50 nM QDs-DNA probe (DNA sequence: 5’-CGGATGGAGTTGTCGGTGTAGATTCATCCGAAA(Dual SH)-3’) in serum-free cell media for 3 hours at 37 °C. Subsequently the serum-free cell media was replaced with fresh FBS-containing cell media and the cells were further incubated for 12 hours. The above cells incubated for 3 and 15 hours were washed twice with 1 × PBS, and then incubated with 200 μL Lysotracker Green DND-26 solution (1: 800 diluted in serum-free cell culture medium) at 37 °C for 30 min, and washed three times with 1 × PBS. Cell images were acquired on a Leica TCS SP5II cofocal laser scanning microscope using a 63 × oil immersion objective. QDs-MB and Lysotracker Green DND-26 were excited at 405 nm and 476 nm respectively, and the emission signals were collected between 600-700 nm and 500-550 nm respectively.

MTT assay.

Cells were cultured as described above and aliquots (100 μL) were seeded (2×10^4 cells) into a 96-well plate. After grown for 24 h, cells were washed with 1× PBS and placed in fresh serum-free cell media. Cells were incubated with QDs (100 nM), TMB (100 nM) and Ce6 (1 μM) for 3 h at 37 °C respectively, washed 3 times with 1× PBS, and then incubated in fresh media for 12 h. PDT was implemented with 365 nm UV light irradiation for 20 min. Afterwards the cells were allowed to continuously grow for 24 h. MTT (5 mg/ml) was added into each well and incubated for 4 hours. Formazan crystals were dissolved in 100 μL DMSO with gentle agitation for 15 min. Cells without any treatment were used as negative control. The absorbance of formazan at 490 nm was measured with a TECAN Infinite M200 PRO plate reader.

Tumor model, animal imaging, and PDT.

All animal experiments were carried out in compliance with institutional animal use and care regulations, Soochoow University. MDA-MB-231 or MCF-7 cells (1×10^7) mixed with BD Matrigel (1:1 v/v) were subcutaneously inoculated in the 4-6 week old nude mice. As the tumors grew up to a diameter of 4-5 mm, the mice were used for imaging and PDT treatment. 0.1 nmol TMB was injected into the tumor by intratumor injection. In vivo fluorescence images were recorded before and after injection (at different time points) on a Maestro In-Vivo Imaging System (Caliper Life Science) (setting: excitation wavelength = 455 nm, emission wavelength = 610-750 nm).

Twenty mice were randomized into five groups (five mice per group) to determine tumor growth rate after the following treatments: (a) 1× PBS; (b) TMB (0.1 nmol); (c) Ce6 (1 nmol); (d) QDs (0.1 nmol). 4 hours after intratumor injection, the tumor was irradiated for 1 hour under 455 nm light. Tumor growth curves of each group were recorded by measuring tumor sizes in two dimensions using a vernier caliper. Tumor was sized once a day, and the tumor volume in cubic mm was approximated by the formula $V(tumor) = (width)^2 \times length/2$.

Biodistribution measurements

0.1 mL of 0.2 nmol QD-DNA nanoprobe (DNA sequence: 5'-CGGATGGAGTTGTCGGTGTAGATTCATCCGAAA(Dual SH)-3') was injected into each nude mouse intravenously via tail vein. The mice were sacrificed after 4 hours and the organs were collected and weighed. Five mice were examined. The organs were digested with 70% nitric acid (3 mL) by heating at 90 °C for 3 hours. The insoluble residues were removed by passing the solutions through a 0.22 μm cellulose filter. The digested samples were diluted with pure H_2O (7 mL). The amount of elemental cadmium in each sample was determined using an ICP Optical Emission Spectrometer

(Varian 710-ES). A dilution series of QDs solutions with known concentrations were measured to generate a calibration curve.

Histological analysis

0.1 mL of 0.2 nmol QD-DNA nanoprobes (DNA sequence: 5'-CGGATGGAGTTGTCGGTGTAGATTCATCCGAAA(Dual SH)-3') were intravenously injected into each mouse via tail vein. The mice were given food and water ad libitum and housed in a 12 h /12 h light/dark cycle before sacrificed after one month. Healthy mice without QDs treatment were used as control group. Five representative organs (heart, liver, spleen, lung, and kidney) were removed, embedded in paraffin, and then sectioned, and finally stained with hematoxylin and eosin. The treated tissue slices were observed and imaged using an optical microscope (Olympus CKX31).

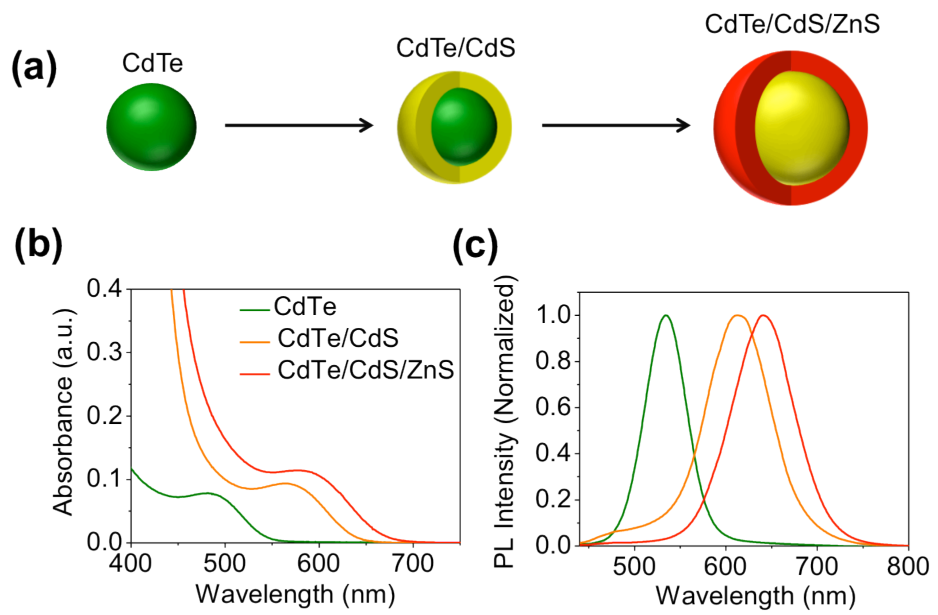

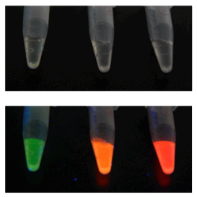

(e)

QDs sample	λ_{ABS}/nm	λ_{EM}/nm	FWHM	QY/%
CdTe	**487**	**535**	**55.5**	**9.7**
CdTe/CdS	**565**	**614**	**81.6**	**39.5**
CdTe/CdS/ZnS	**580**	**643**	**79.6**	**44.7**

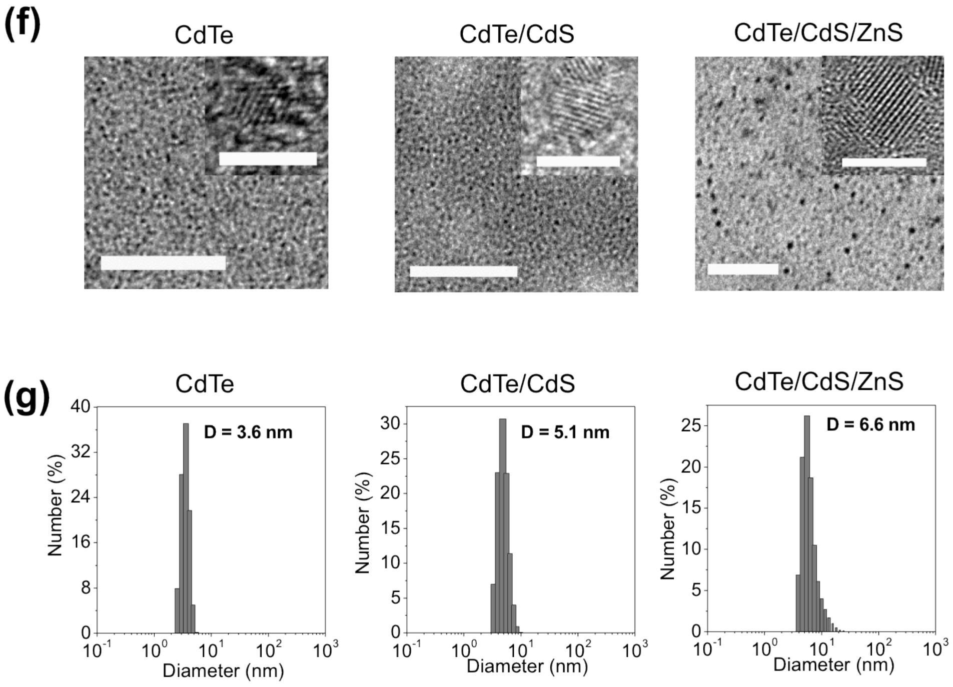

Figure S1. Characterization of CdTe, CdTe/CdS, and CdTe/CdS/ZnS QDs. (a) Schematic illustration of the synthetic route of CdTe/CdS/ZnS QDs. (b) Absorption spectra and (c) photoluminescence

spectra of CdTe, CdTe/CdS, and CdTe/CdS/ZnS QDs. (d) Photographs of CdTe, CdTe/CdS, and CdTe/CdS/ZnS QDs solutions under ambient light (top) and UV light (bottom). (e) Summary of the first absorption peak wavelength, PL peak wavelength, full width at half maximum (FWHM) of PL peak, and quantum yield of CdTe, CdTe/CdS, and CdTe/CdS/ZnS QDs. (f) Low magnification and high-resolution (inset) TEM images of CdTe, CdTe/CdS, and CdTe/CdS/ZnS QDs. (scale bars are 50 nm and 5 nm respectively) (g) Hydrodynamic diameters of CdTe, CdTe/CdS, and CdTe/CdS/ZnS QDs measured by DLS.

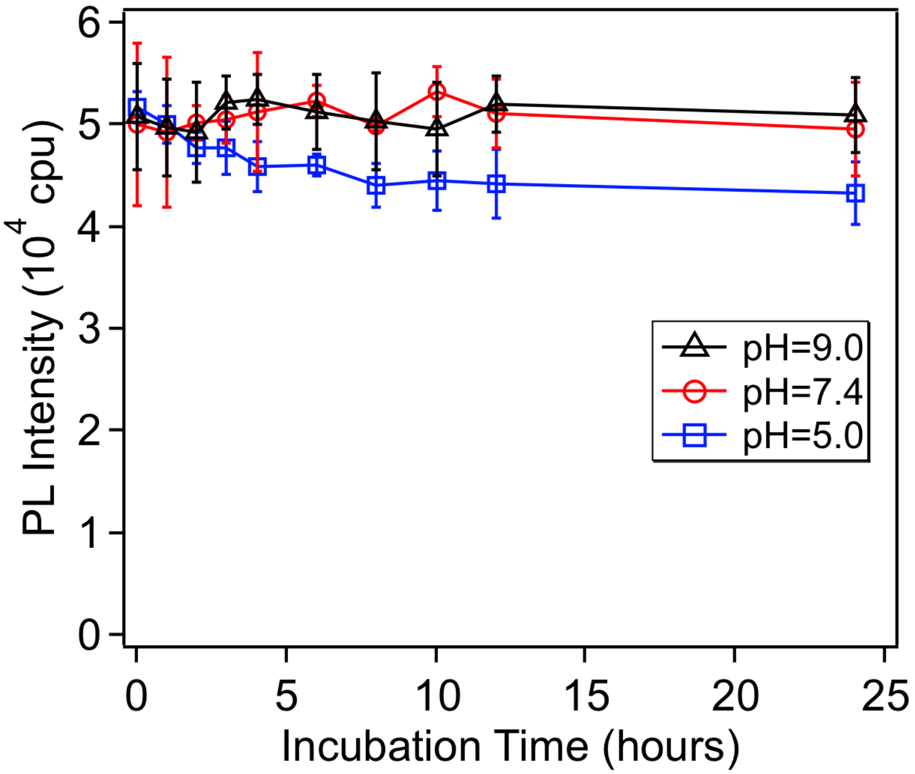

Figure S2. PL intensity of CdTe/CdS/ZnS QDs incubated in buffers with different pH values: HAc-NaAc (pH=5.0), Tris-HCl (pH=7.4), and NH_4HCO_3-$(NH_4)_2CO_3$ (pH=9.0).

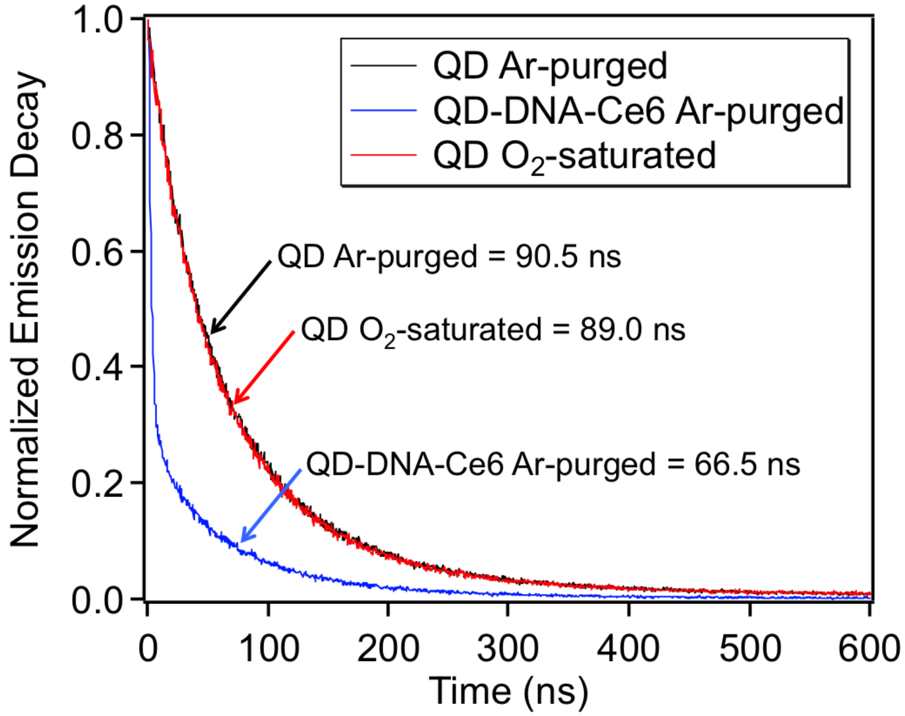

Figure S3. Fluorescence lifetime decay curves of unconjugated QDs in Ar-purged solution (black) and O_2-saturated solution (red) and QD-DNA-Ce6 conjugates in Ar-purged solution (blue).

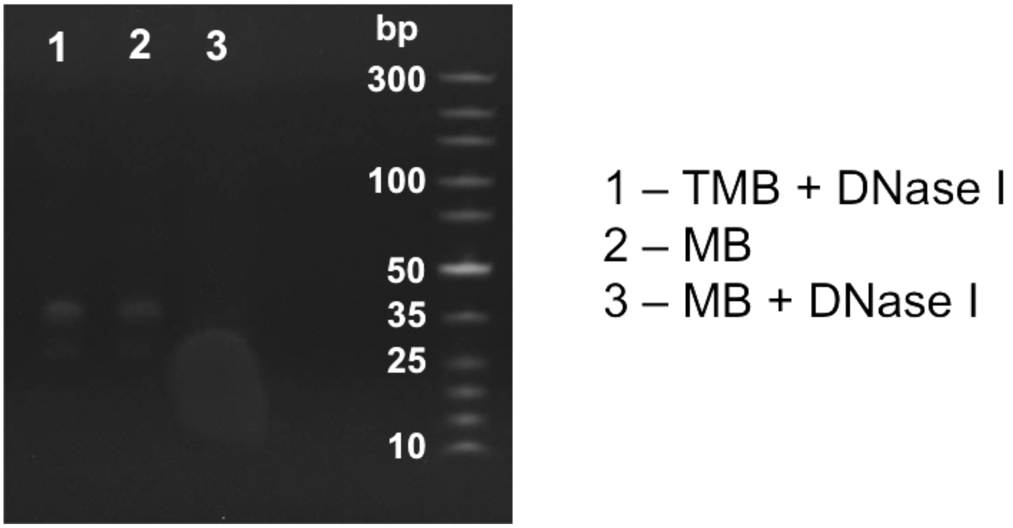

Figure S4. Stability of TMB and MB after DNase I treatment. DNase I treated TMB was incubated in HCl (pH=3) to digest QDs before electrophoresis. MB without and with DNase I treatment was used as negative and positive controls respectively.

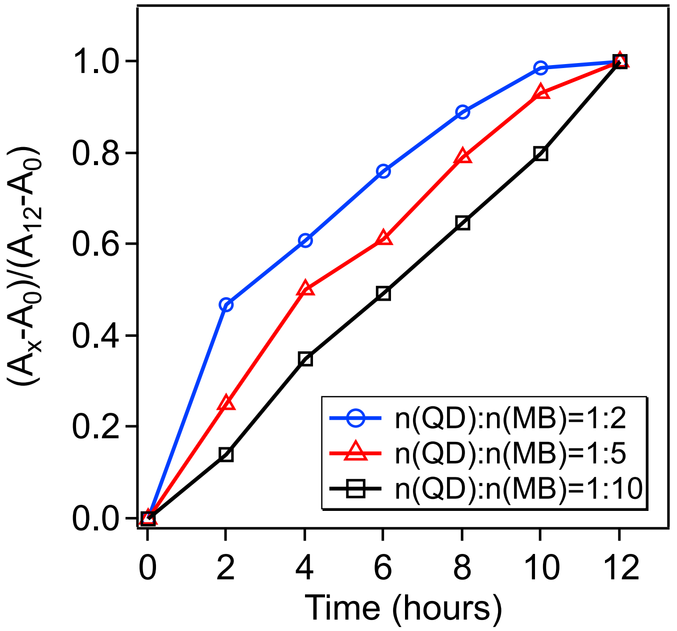

Figure S5. Activation kinetics of TMB constructed with different QD/MB molar ratio. The TMB was incubated with complementary DNA (Cyclin D1 sequence) for 12 hours and the PL spectra were recorded every two hours. A_x stands for the area of integrated PL spectrum at the time point of x hours.

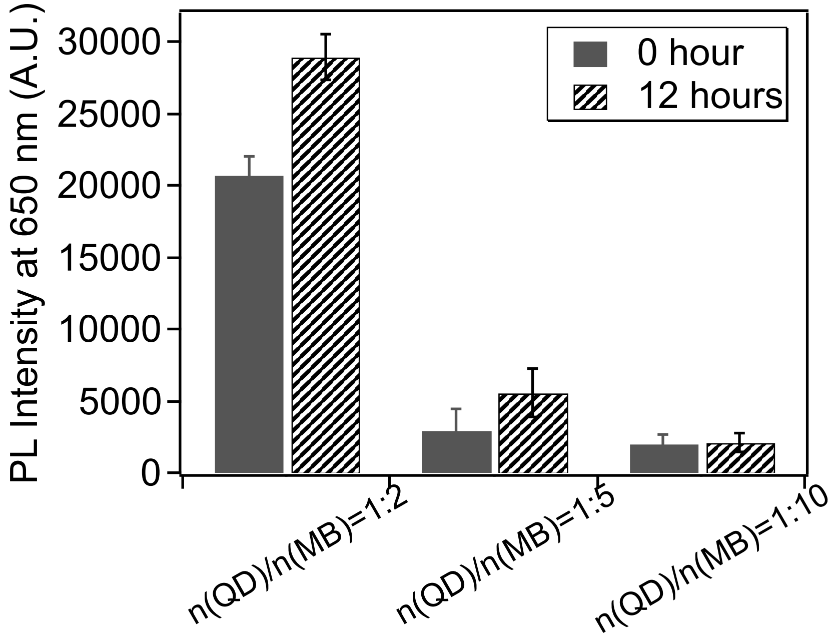

Figure S6. Nuclese degradation of TMB as a function of QD/MB molar ratio. More efficient quenching of QD PL and nuclease protection could be achieved with an increasing MB to QD ratio.

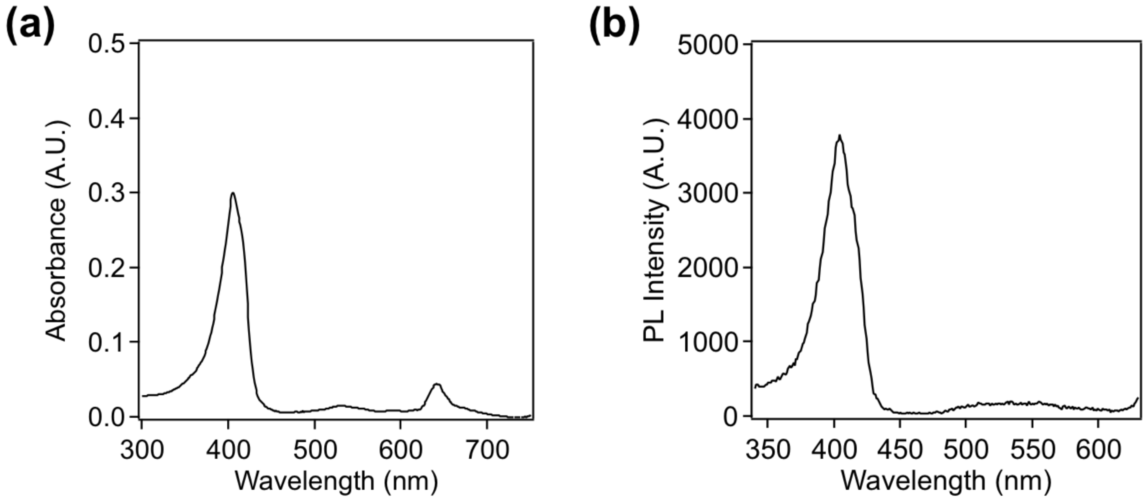

Figure S7. (a) Absorption spectrum of Ce6 molecules. (b) Excitation spectrum of Ce6 molecules with the emission wavelength set to 656 nm.

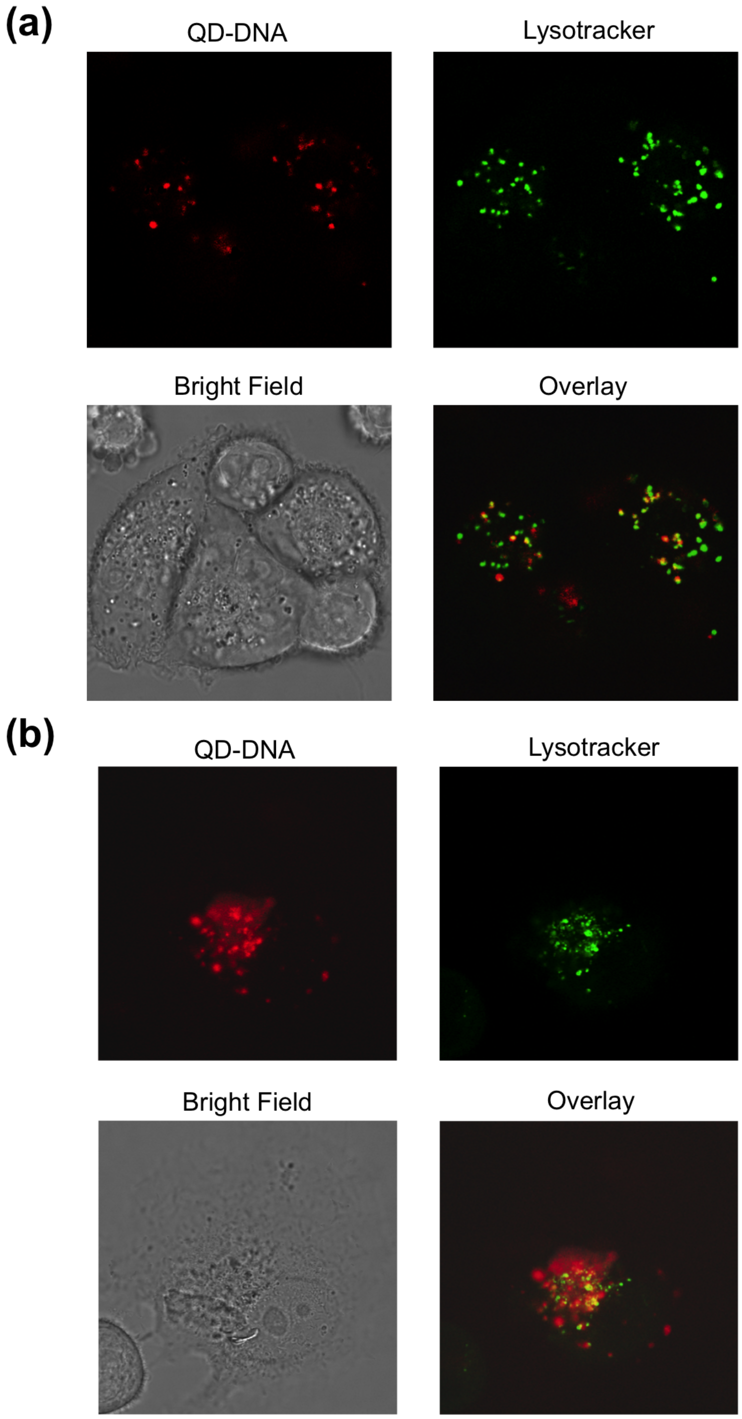

Figure S8. Confocal and co-localization images of QD-DNA probes incubated with MCF-7 cells for 3 hours (a) and 15 hours (b). In order to track the QD signal, the BHQ3 quencher was not conjugated to the probe. The QD fluorescence was shown in red and the lysotracker fluorescence was shown in green.

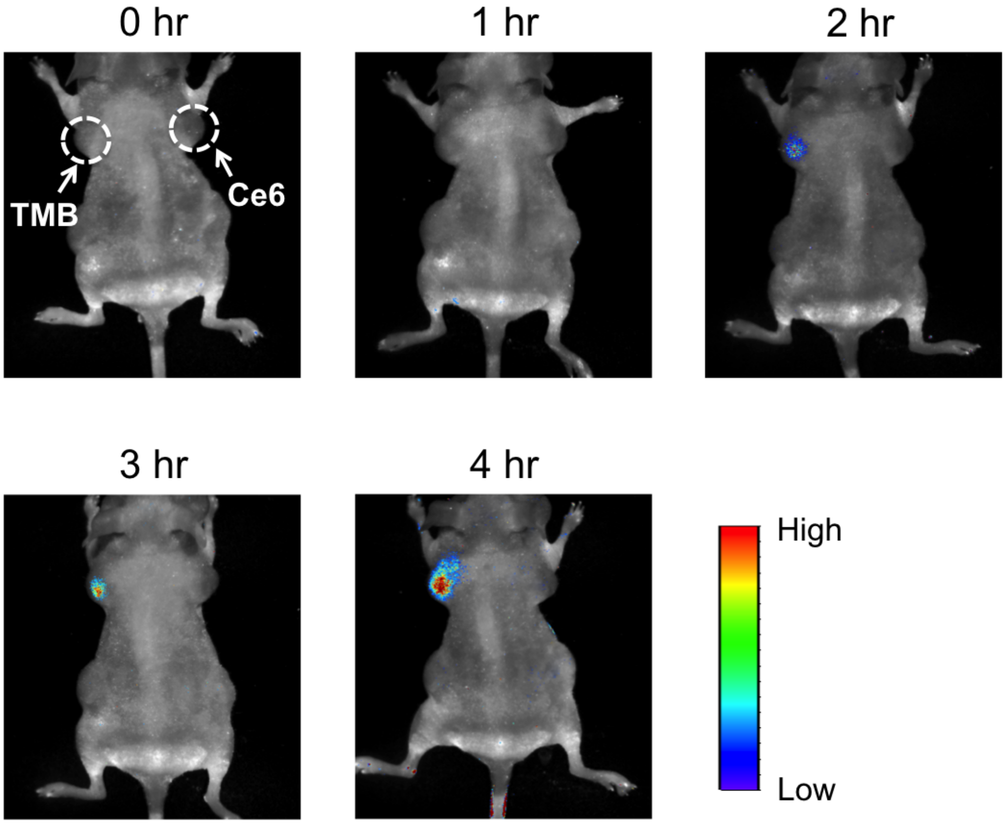

Figure S9. Whole body fluorescence images of MCF-7 tumor-bearing mouse injected with TMB and Ce6 (same Ce6 concentration). The images were recorded pre-injection (0 hour) and post-injection at different time points (1, 2, 3, 4 hours). Images were acquired with 455 nm excitation and the emission signals were collected between 610 and 750 nm.

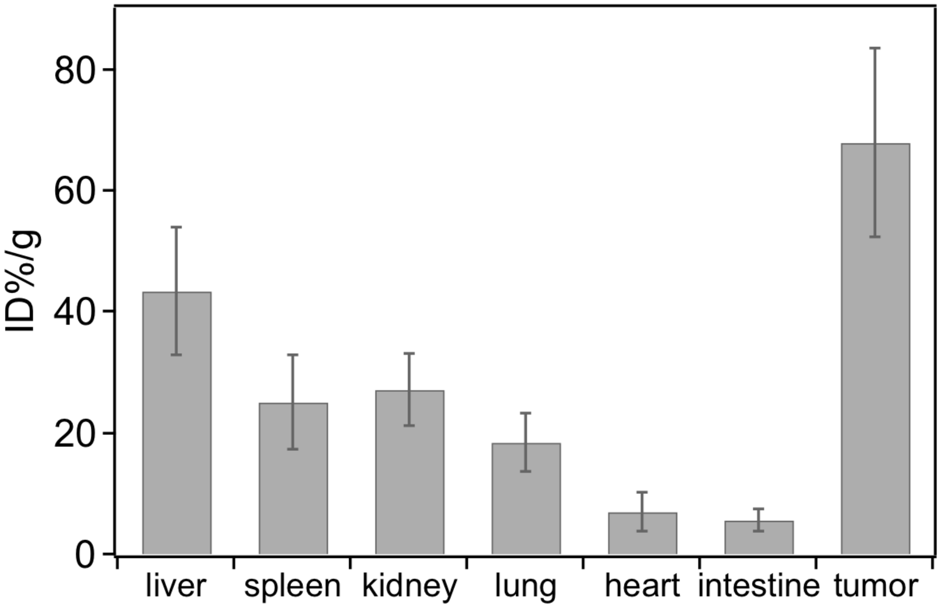

Figure S10. Quantitative analysis of biodistribution of QD-DNA nanoprobes using ICP-AES. Five nude mice were injected with QD-DNA nanoprobes via tail vein and were sacrificed after 4 hours. The biodistribution of QDs in each organ is calculated to be: liver (43.5%ID/g), spleen (25.2%ID/g), lung (18.6%ID/g), kidney (27.3%ID/g), heart (7.2%ID/g), intestine (5.8%ID/g), and tumor (68.1%ID/g)

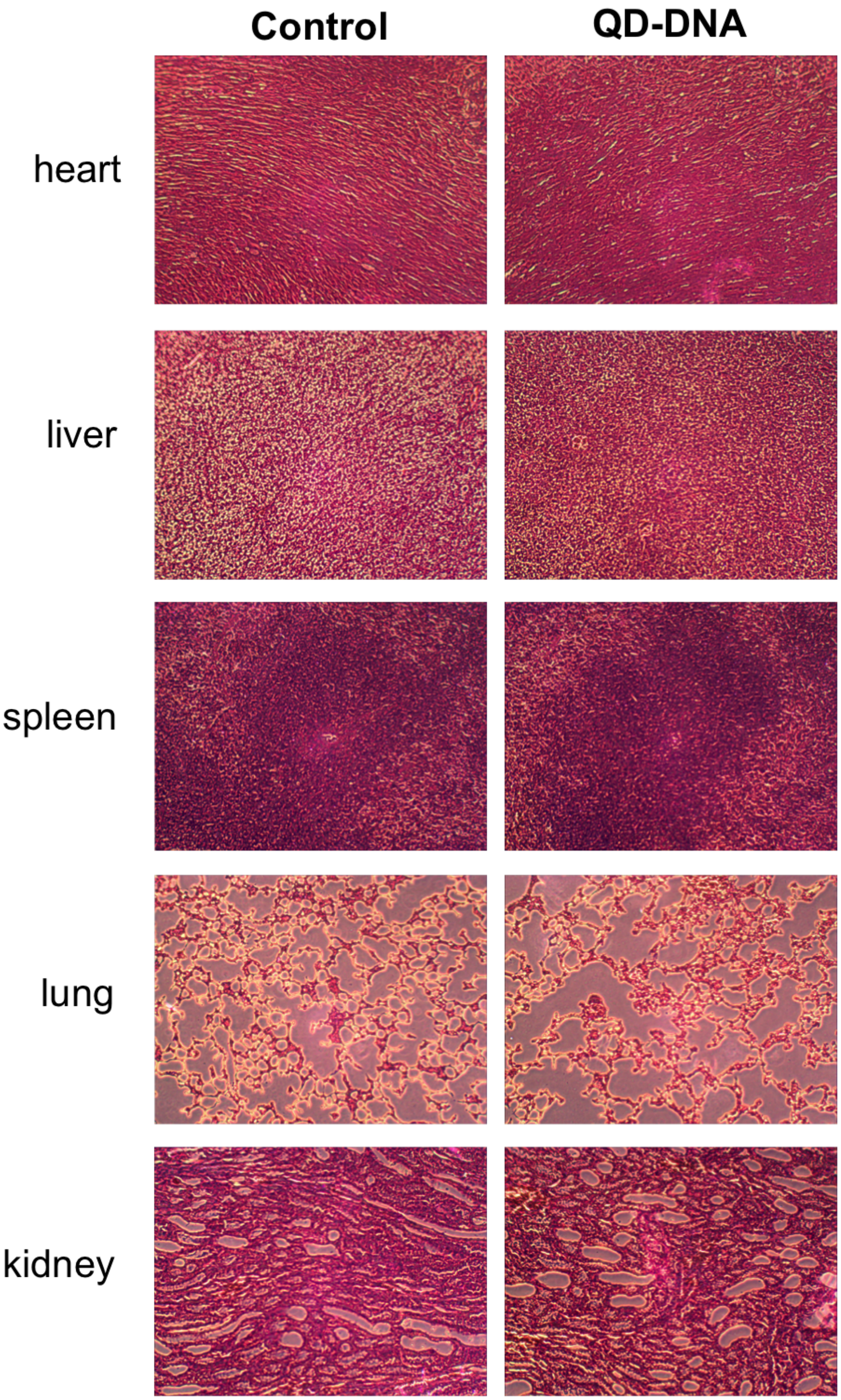

Figure S11. Representative organ histology H&E staining images of the mouse injected with QD-DNA nanoprobes and untreated healthy control mouse. The QD-DNA nanoprobes are not destructive to all the examined organs.
